# Clinicopathological analysis and prognostic significance of programmed cell death-ligand 1 protein and mRNA expression in non-small cell lung cancer

**DOI:** 10.1371/journal.pone.0198634

**Published:** 2018-06-01

**Authors:** Hyojin Kim, Hyun Jung Kwon, Soo Young Park, Youngmi Park, Eunhyang Park, Jin-Haeng Chung

**Affiliations:** 1 Department of Pathology, Seoul National University Bundang Hospital, Seongnam, Republic of Korea; 2 Medical Research Collaborating Center, Seoul National University Bundang Hospital, Seongnam, Republic of Korea; 3 Department of Pathology, Seoul National University College of Medicine, Seoul, Republic of Korea; University of South Alabama Mitchell Cancer Institute, UNITED STATES

## Abstract

In this study, we present the clinicopathological features associated with PD-L1 protein and mRNA expression in a large Asian cohort of patients with non-small cell lung cancer (NSCLC) and assessed the prognostic implications of PD-L1 expression, particularly in early stage NSCLC. We retrospectively analyzed 687 NSCLC specimens (476 adenocarcinoma and 211 squamous cell carcinoma) using tissue microarray. PD-L1 immunohistochemistry (IHC) was performed using Dako 22C3 pharmDx assay and *PDL1* mRNA was measured using RNA in situ hybridization (RISH). The overall prevalence of PD-L1 protein expression was 25.2% in tumor cells and *PDL1* mRNA expression was 11.9%. There was a strong positive correlation between PD-L1 IHC and RISH results (Spearman’s rho = 0.6, p<0.001). In adenocarcinoma, PD-L1 protein and mRNA expressions significantly correlated with poorly differentiated histologic subtype (p<0.001 and p = 0.002, respectively). PD-L1 expression was also associated with genetic alteration in adenocarcinoma. High PD-L1 expression level was associated with *EGFR*-naïve and *KRAS*-mutant subgroup (p = 0.001 and p = 0.017, respectively). With a 1% cut-off value, PD-L1 protein expression showed a short overall survival duration in early stage adenocarcinoma with marginal significance (p = 0.05, Hazard ratio = 1.947). Our study revealed that PD-L1 expression varied with histologic subtype and genomic alteration status in lung adenocarcinoma, and activation of the PD-L1 pathway may be a poor prognostic factor especially in early stage lung adenocarcinoma. In addition, *PDL1* RISH showed promising results in predicting PD-L1 protein expression in NSCLC.

## Introduction

Researchers have recently become interested in developing immunotherapies for the treatment of non-small cell lung cancer (NSCLC), particularly monoclonal antibodies targeting the programmed cell death-1 (PD-1) receptor and its ligand (PD-L1) [1, 2]. Interaction of PD-1 with PD-L1 inhibits T-cell activation, allowing tumor cells to bypass immune surveillance. Therefore, blockade of the PD-1/PD-L1 axis may enhance the active immune response against tumors. Currently, different types of monoclonal antibodies targeting PD-1 or PD-L1, including nivolumab for NSCLC with squamous cell histology [3] and non-squamous cell histology [4] in the second-line setting, pembrolizumab for NSCLC with high PD-L1 expression (≥ 50%) in the first-line setting [5] or in the second-line setting for tumors with 1–49% PD-L1 expression [6], and atezolizumab for all subtypes of NSCLC in the second-line setting [7], are available. Responses to PD-1/PD-L1 inhibitors are improved in patients with high tumor PD-L1 expression compared with those exhibiting low or no PD-L1 [4–6,8]. Therefore, PD-L1 protein expression is the only biomarker that can predict which patients are more likely to respond to anti-PD-1/PD-L1 therapy in the clinical setting. However, the correlations between PD-L1 expression in tumor cells and treatment response to anti-PD-1 or anti-PD-L1 therapy is still unclear because almost 10% of patients with PD-L1-negative tumors also responded to PD-1/PD-L1 inhibitors in the above clinical trials [4,6].

Besides acting as a predictive biomarker, PD-L1 shows inconsistent results among various studies as a prognostic biomarker. Studies investigating the prognostic role of PD-L1 and its association with clinicopathological features and driver mutations in NSCLC have yielded quite different results [9–13]. This discrepancy may be attributed to differences in ethnicity, heterogeneous histological subtypes, and stages. Furthermore, clinical trials with checkpoint inhibitors have focused on advanced, inoperable tumors; thus, data reporting the predictive and prognostic roles of PD-L1 expression in early-stage NSCLC are limited.

The variety of PD-L1 immunohistochemical (IHC) assays, involving the use of different antibodies and interpretation criteria, may also contribute to the lack of consistent results [14]. Given the difficulties associated with PD-L1 IHC, an alternative method for accurately evaluating PD-L1 expression is needed. An antibody-independent assay for RNA in situ hybridization (RISH) in formalin-fixed, paraffin-embedded (FFPE) tumor tissues using an RNAscope assay has been favored for its specificity and interpretative objectivity. In gastric cancer and small cell lung cancer, *PDL1* mRNA exhibited a positive nonlinear relationship with PD-L1 protein using this assay, suggesting the potential applications of the RNAscope assay in future clinical studies [15,16].

In this study, we evaluated the clinicopathological features associated with PD-L1 protein and mRNA expression in a large Asian cohort of patients with NSCLC and investigated the prognostic implications of PD-L1 expression, particularly in early stage NSCLC.

## Materials and methods

### Patients and samples

Our cohort consisted of 687 patients with NSCLC, including 476 with adenocarcinoma (ADC) and 211 with squamous cell carcinoma (SqCC) who underwent surgical resection between May 2003 and December 2012 at Seoul National University Bundang Hospital. None received pre-operative chemotherapy or radiation therapy. Clinicopathological information was obtained from clinical records and pathology reports. The pathologic staging was based on the 7^th^ edition of the American Joint Committee on Cancer staging manual [17]. The study protocol was approved by the Institutional Review Board of Seoul National University Bundang Hospital (B-1704/393-303).

### Histological analyses

All resected tumor specimens were fixed with formalin and then stained with hematoxylin and eosin (H&E). All H&E slides were carefully reviewed by two of the authors (H. Kim and J.H. Chung) to confirm the original diagnosis and classify the histological subtype. ADC in situ and minimally invasive ADC samples were excluded from the study. All other invasive ADC samples were categorized as lepidic, papillary, acinar, micropapillary, solid, or invasive mucinous according to the 2015 World Health Organization Classification of Lung Tumors [18]. These histological subtypes were used to determine tumor grade (lepidic, well differentiated; acinar and papillary, moderately differentiated; and micropapillary and solid, poorly differentiated).

### Construction of the tissue microarray (TMA)

The slides were independently reviewed by two pathologists (H. Kim and J.H. Chung) to select the most representative sections. The most representative tumor area was carefully marked on the H&E-stained slide of each sample tissue. A TMA was constructed using 2-mm-diameter cores derived from the representative tumor areas selected at random of the FFPE tissue blocks from each case by SuperBioChips Laboratories (Seoul, Korea).

### IHC analysis of PD-L1 protein

TMAs were sectioned at a thickness of 4-μm and stained using the Dako pharmDx assay. Briefly, the slides were stained with anti-PD-L1 22C3 mouse monoclonal primary antibodies with the EnVision FLEX visualization system on a Dako Autostainer Link 48 instrument (Carpinteria, CA, USA), along with negative control reagents and cell line run controls, as per the manufacturer’s instructions. The IHC slides were scored independently by two pathologists (H.J. Kwon and H. Kim). PD-L1 was considered positive in tumor cells only in cases of at least 100 viable tumor cells, if membranous staining alone or membranous and cytoplasmic staining together was present. Membranous staining in tumor cells directly adjacent to immune cells was not considered positive if the surface touching immune cells was the only stained part. The percentage of stained cells in the overall area of the tumor (Tumor Proportion Score) was scored regardless of intensity [6]. Cases were then classified by two different cut-off values, 1% and 50%, based on the published association of this cut-off with anti-PD-1 therapeutic response [6].

### RNA *in situ* hybridization of *PDL1* mRNA

*PDL1* mRNAs were measured using RNAscope assays (Advanced Cell Diagnostics [ACD], Hayward, CA, USA) following the manufacturer’s instructions [19]. Briefly, 5-μm-thick sections were deparaffinized; incubated with pretreatment reagents 1, 2, and 3 at room temperature for 10 min; boiled for 15 min; and incubated at 40°C for 30 min. TMA sections were then hybridized with Hs-CD274-probes (ACD) at 40°C for 2 h. Hybridization signals were amplified and visualized with an RNAscope 2.0 HD detection kit (Red). RNAscope results were examined under a standard bright field microscope at 200–400× magnification. Positive signals presented as red punctuate dots. PPIB and DapB were used as positive and negative probes, respectively, to control tissue RNA conditions and nonspecific hybridization.

*PD-L1* mRNA signals were in the tumor compartment or mesenchyme, as visualized by red dotted or clustered patterns. No standard scoring criteria for *PD-L1* mRNA expression in NSCLC had been determined; therefore, we adopted the RNAscope system scoring guidelines (“RNA scope score”): 0 (no staining or < 1 dot per 10 cells); 1 (1–3 dots per cell); 2 (4–9 dots per cell); 3 (10–15 dots per cell); and 4 (> 15 dots per cell and > 10% dots in clusters) [19]. We also evaluated the tumor proportion that showed at least 1 dot. We classified signals according to the proportion as follows: 0 (0 and < 1%); 1 (1–9%); 2 (10–49%); and 3 (50–100%), which was defined as the “RNA proportion score”. Because *PD-L1* RNA scope and proportion scores showed a linear correlation (r = 0.83, *p* < 0.01, data not shown), cases showing either an RNA scope score of 1 or more or an RNA proportion score of 1 or more were designated as *PD-L1* mRNA positive.

### Detection of mutations in *EGFR* and *KRAS* and rearrangement of the *ALK* gene

Polymerase chain reaction and DNA sequencing with FFPE tissue samples were used to analyze *EGFR* mutations in exons 18–21 and *KRAS* mutations at codons 12, 13, and 61, as described previously [20]. Rearrangement of the *ALK* gene was assessed using fluorescence in-situ hybridization with an ALK probe (Vysis LSI ALK Break Apart Rearrangement probe; Abbott Molecular, Park, IL, USA) and a 15% cut-off value, as described previously [20].

### Statistical analysis

Statistical analysis was carried out using Stata Statistical Software version 14 (Stata Corp., College Station, TX, USA) and R program (R Foundation for Statistical Computing, Vienna, Austria). Spearman’s test and logistic regression were performed to compare assays and determine appropriate cut-off values. Cohen’s ĸ coefficient of agreement was obtained to cross-check the results. A Kaplan-Meier analysis was performed to construct survival curves, and statistical significance was assessed using log-rank tests. A multivariate analysis was performed by Cox proportional hazards regression modeling. All statistical tests were two sided, and statistical significance was accepted for *p* values of less than 0.05.

## Results

### Clinicopathological characteristics

Clinicopathological characteristics are summarized in Table 1. Briefly, there were a total of 429 men (62.4%) and 258 women (37.6%) with a median age of 64 years (range: 21–85 years). Approximately half of the patients were never smokers (n = 297; 43.2%). This may be the reason that ADC was the most prevalent histological subtype (n = 476; 69.3%). The pathological stage was I in 359 patients (52.2%), II in 162 patients (23.6%), III in 141 patients (20.6%), and IV in 25 patients (3.6%).

**Table 1:** Clinicopathological characteristics.

		Adenocarcinoma		Squamous cell carcinoma		Total	
Characteristic		Number of cases	%	Number of cases	%	Number of cases	%
**Age (year)**							
	Median (range)	64 (21–83)		68 (31–85)		64 (21–85)	
**Sex**							
	Male	229	48.1	200	94.8	429	62.4
	Female	247	51.9	11	5.2	258	37.6
**Smoking status**^¶^							
	Never smoker	284	59.7	13	6.2	297	43.2
	Current smoker	91	19.1	122	57.8	213	31.0
	Ex-smoker	101	21.2	76	36.0	177	25.8
**Tumor size (cm)**							
	Mean (range)	3.1 (0.5–16.0)		4.0 (0.8–14.5)		3.4 (0.5–16.0)	
**Pleural invasion**							
	Absent	272	57.1	149	70.6	421	61.3
	Present	204	42.9	62	29.4	266	38.7
**Venous invasion**							
	Absent	361	75.8	168	79.6	529	77.0
	Present	115	24.2	43	20.4	158	23.0
**Lymphatic invasion**							
	Absent	248	52.1	130	61.6	378	55.0
	Present	228	47.9	81	38.4	309	45.0
**Pathologic stage**							
	I	271	56.9	88	41.7	359	52.5
	II	90	18.9	72	34.1	162	23.6
	III	96	20.2	45	21.3	141	20.6
	IV	19	4.0	6	2.9	25	3.6
**PD-L1 protein expression**							
	< 1%	399	83.8	115	54.5	514	74.8
	1–49%	48	10.1	57	27.0	105	15.3
	≥ 50%	29	6.1	39	18.5	68	9.9
**PDL1 mRNA expression**							
	Negative	447	93.9	158	74.9	605	88.1
	Positive	29	6.1	53	25.1	82	11.9
**Total**		476	69.3	211	30.7	687	100

PD-L1, programmed cell death ligand-1; mRNA, messenger RNA

^**¶**^ smoking status was defined as follows: never smoker (<100 cigarettes per lifetime); current smoker (≥100 cigarettes per lifetime and smoked at the time of lung cancer diagnosis or quit ≤1 year prior to the diagnosis); ex-smoker (≥100 cigarettes per lifetime and quit >1 year prior to the diagnosis)

### PD-L1 protein and mRNA expression

**Fig 1:**
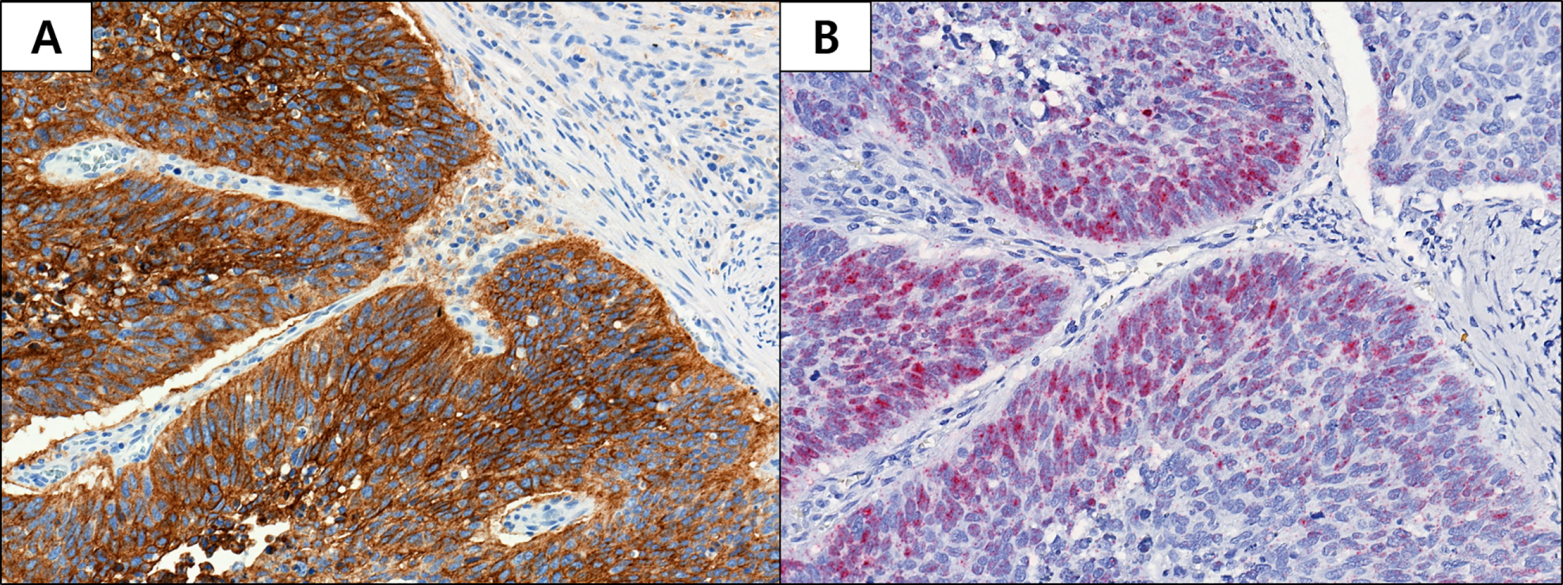
**Programmed Death Ligand 1 (PD-L1) Protein (A) and mRNA (B) Expression in Non-small Cell Lung Cancer.** (A) Membranous expression of PD-L1 protein in tumor cells (20× magnification). (B) *PD-L1* mRNA signals located in the nucleus and mesenchyme within tumor compartments are denoted by red dotted or clustered patterns (20× magnification).

The overall prevalence of PD-L1 protein expression in tumor cells was 25.2% (173/687; Table 1). With a 1% cut-off, PD-L1 was positive in 16.2% (77/476) and 45.5% (96/211) of ADC and SqCC, respectively. With a 50% cut-off, the positive rates were 6.1% (29/476) for ADC and 18.5% (39/211) for SqCC. Thus, PD-L1 protein appeared to be present in a higher percentage of SqCC samples than ADC samples (*p* < 0.05). *PD-L1* mRNA expression was detected in 11.9% (82/687) of patients. In subgroup analysis, SqCC showed higher mRNA positivity than ADC (25.1% versus 6.1%; *p* < 0.05). Fig 1 shows representative images of PD-L1 protein (Fig 1A) and mRNA expression (Fig 1B) based on IHC and RNAscope, respectively. Membranous expression of PD-L1 IHC can be readily seen in A, while in B, red dotted or clustered PDL1 mRNA signals can be noted.

### Correlation between PD-L1 protein and mRNA expression

PD-L1 protein expression showed a strong positive correlation with *PD-L1* mRNA expression (Spearman’s rho = 0.6, *p* < 0.001). As the TPS of PD-L1 protein expression increased, PDL1 mRNA expression was observed frequently (Table 2).

**Table 2:** Correlation between PD-L1 mRNA and protein expression.

IHC TPS (%)	mRNA expression (number, %)		Total
	Negative	Positive	
**<1**	500 (97.3)	14 (2.7)	514 (74.8)
**1–49**	85 (80.9)	20 (19.1)	105 (15.3)
**≥50**	20 (29.4)	48 (70.6)	68 (9.9)
**Total**	605 (88.1)	82 (11.9)	687 (100)

IHC, immunohistochemistry; TPS, tumor proportion score

We calculated the overall percentage agreement (OPA) pairwise between assays at two PD-L1 IHC cut-off values (1% and 50%). OPA with 1% and 50% IHC cut-off values were 80.1% and 91.1%, respectively (Table 3). Positive and negative percentage agreement (PPA and NPA) were calculated for mRNA assays against the IHC (1% and 50% cut-off values). With a 1% cut-off, the PPA and NPA of mRNA assays were 78.1% and 80.3%, respectively. Applying a 50% cut-off, PPA was decreased (46.9%), whereas NPA was increased (95.7%; Table 3).

**Table 3:** Agreement between PD-L1 protein and mRNA expression results.

	PD-L1 IHC 1% cutoff			PD-L1 IHC 50% cutoff		
	OPA	PPA	NPA	OPA	PPA	NPA
***PDL1* mRNA**	80.1%	78.1%	80.3%	91.1%	46.9%	95.7%

PD-L1, Programmed cell death ligand-1; IHC, immunohistochemistry; mRNA, messenger RNA; OPA, overall percentage agreement; PPA, positive percentage agreement; NPA, negative percentage agreement

### Association between PD-L1 status and clinicopathological parameters

**Fig 2:**
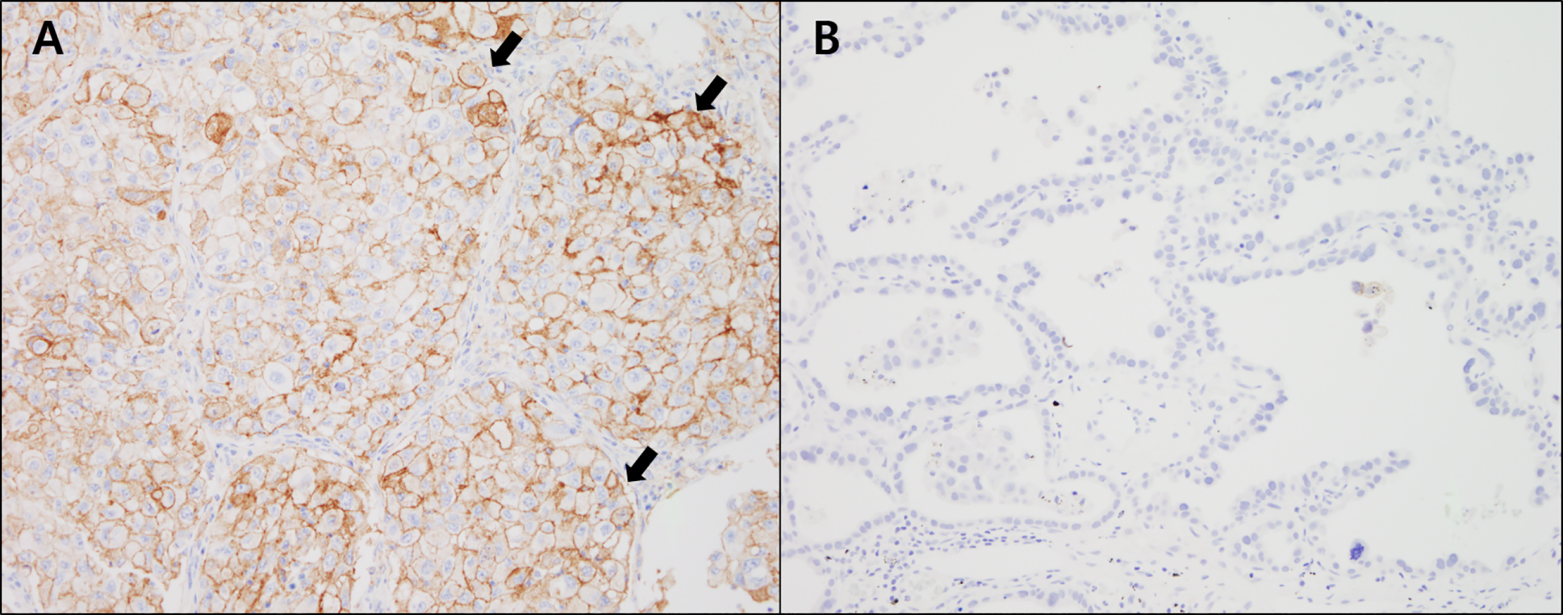
Microphotographs of representative examples of PD-L1 protein expression according to histological subtypes of lung adenocarcinoma. (A) PD-L1 protein is expressed in tumor cell membranes (>50%) in 37.3% of solid predominant ADC (arrowheads). (B) In contrast, PD-L1 was not expressed in most lepidic predominant ADC (97.6%). (A and B, 20× magnification).

Next, we investigated the associations between PD-L1 status and clinicopathological parameters in ADC and SqCC. In ADC, both PD-L1 protein and mRNA expression were correlated with histologic subtype (*p* < 0.001 and *p* = 0.002, respectively; Table 4). PD-L1 showed higher expression in the poorly differentiated (solid and micropapillary predominant) histologic subgroup than in the well-differentiated (lepidic predominant) subgroup (Fig 2). PD-L1 expression was also associated with genetic alterations. Although PD-L1 protein and mRNA expression levels were lower in the *EGFR*-mutated group than in the *EGFR*-negative group (*p* = 0.001 and *p* = 0.016, respectively), PD-L1 protein expression was higher in the *KRAS*-mutated group than in the *KRAS*-negative group (*p* = 0.017). Smoking history, pathological stage, and *ALK* status were not associated with PD-L1 status. In SqCC, only tumor size was associated with PD-L1 protein expression with marginal significance (*p* = 0.049; data not shown).

**Table 4:** Association between PD-L1 status and clinicopathologic variables in lung adenocarcinoma.

		PD-L1 IHC (number (%))				PD-L1 RNA scope (number (%))		
	Total	Negative	Weak positive	Strong positive	*p* value	Negative	Positive	*p* value
**Smoking history**								
Yes	192	156 (81.3%)	22 (11.5%)	14 (7.3%)	> 0.05	180 (93.8%)	12 (6.2%)	> 0.05
No	284	243 (85.6%)	26 (9.2%)	15 (5.3%)		267 (94.0%)	17 (6.0%)	
**Histologic subtype** ^¶^								
WD	42	41 (97.6%)	1 (2.4%)	0	<0.001	41 (97.6%)	1 (2.4%)	0.002
MD	360	309 (85.8%)	33 (9.2%)	18 (5.0%)		343 (95.3%)	17 (4.7%)	
PD	68	43 (62.7%)	14 (20.9%)	11(16.4%)		57 (83.6%)	11 (16.4%)	
Mucinous	6	6 (100%)	0	0		6 (100%)	0	
**Pathologic stage**								
IA-IIA	340	286 (84.1%)	33 (9.7%)	21 (6.2%)	> 0.05	320 (94.1%)	20 (5.9%)	> 0.05
IIB-IV	136	113 (83.1%)	15 (11.0%)	8 (5.9%)		127 (93.4%)	9 (6.6%)	
***EGFR* mutation**								
Present	223	201 (90.1%)	17 (7.6%)	5 (2.2%)	0.001	216 (96.9%)	7 (3.1%)	0.016
Absent	229	179 (78.2%)	27 (11.8%)	23 (10.0%)		209 (91.3%)	20 (8.7%)	
***KRAS* mutation**								
Present	23	15 (65.2%)	4 (17.4%)	4 (17.4%)	0.017	20 (87.0%)	3 (13.0%)	> 0.05
Absent	237	203 (85.7%)	23 (9.7%)	11 (4.6%)		223 (94.1%)	14 (5.9%)	
***ALK* rearrangement**								
Present	24	21 (87.5%)	1 (4.2%)	2 (8.3%)	> 0.05	24 (100%)	0	> 0.05
Absent	181	161 (89.0%)	12 (6.6%)	8 (4.4%)		177 (97.8%)	4 (2.2%)	

PD-L1, Programmed cell death ligand-1; IHC, immunohistochemistry; WD, well differentiated; MD, moderately differentiated; PD, poorly differentiated; EGFR, Epidermal growth factor receptor; KRAS, Kirsten rat sarcoma 2 viral oncogene homolog; ALK, Anaplastic lymphoma kinase.

^**¶**^ lepidic, well differentiated; acinar and papillary, moderately differentiated; and micropapillary and solid, poorly differentiated.

### Survival analysis

**Fig 3:**
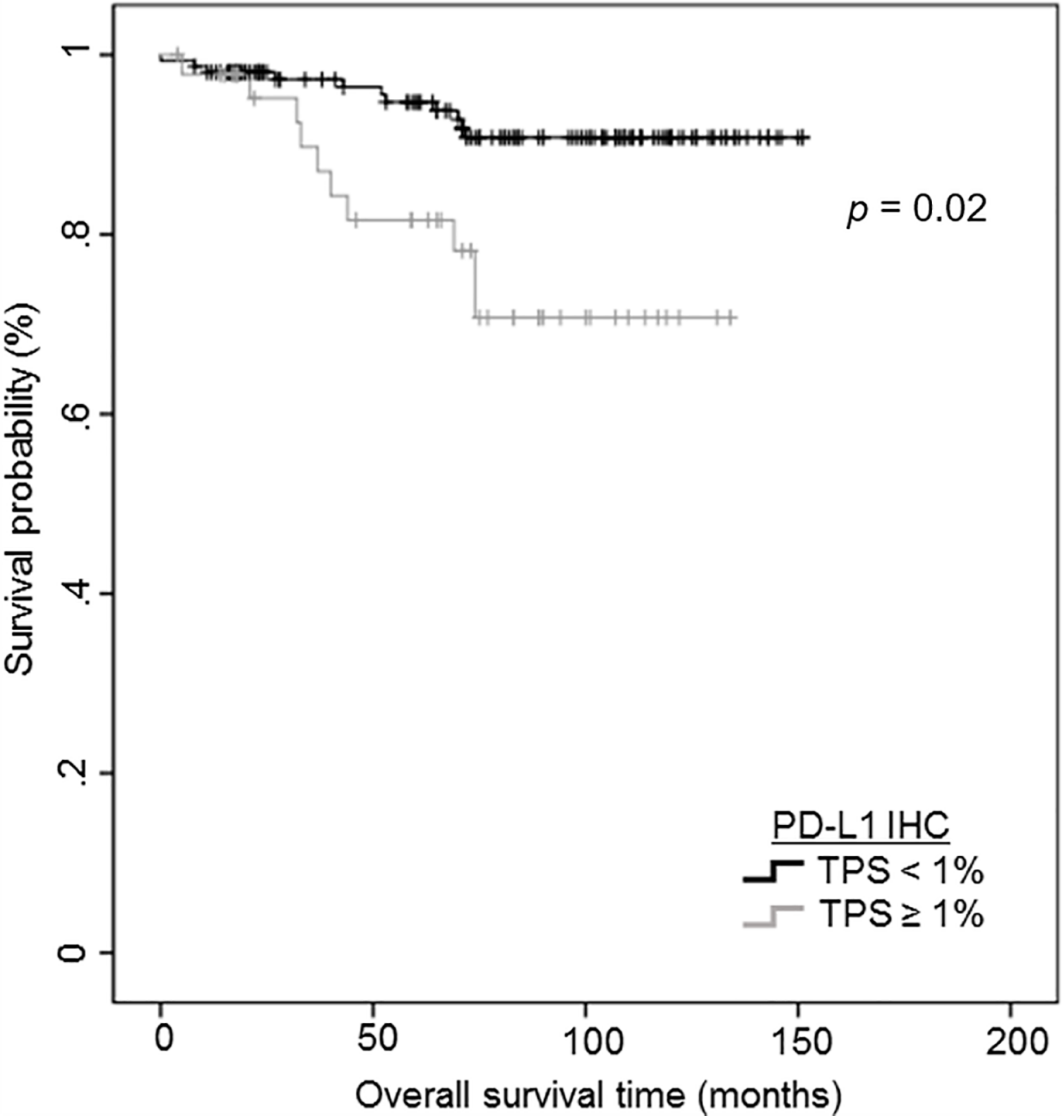
Kaplan–Meier curves depicting the prognostic impact of programmed cell death ligand-1 (PD-L1) protein expression on overall survival in early stage (I and IIA) non-small cell lung cancer subgroup. Cases with PD-L1 protein expression over 1% showing a shorter overall survival duration (*p* = 0.02).

We performed survival analysis to investigate the prognostic role of PD-L1 protein and mRNA expression in ADC and SqCC. In ADC, lymphovascular/perineural invasion and pathologic TNM stage were independent poor prognostic factors for disease-free survival (DFS) and overall survival (OS; Table 5A). PD-L1 expression was not associated with patient survival in the full cohort of patients with ADC. To investigate the prognostic significance of PD-L1 expression, we performed survival analysis in the early stage (I and IIA) ADC subgroup containing 340 patients. In subgroup analysis, PD-L1 protein expression over 1% was associated with shorter OS using univariate analysis (*p* = 0.02) (Fig 3) and tended to show poor prognosis with marginal significance (*p* = 0.05, hazard ratio: 1.947) in multivariate analysis after adjusting for the conventional clinicopathological covariates (Table 5B). For *PD-L1* mRNA, there were no significant survival differences in both the full cohort and early stage ADC subgroup. In SqCC, PD-L1 protein and mRNA expression levels were not associated with survival (data not shown).

**Table 5:** Survival analysis in full cohort (A) and early-stage subgroup (B) of lung adenocarcinoma.

**A (n = 476)**							
		**Disease-free survival**			**Overall survival**		
		**Univariate**	**Multivariate**		**Univariate**	**Multivariate**	
**Clinicopathologic variables**	**Category**	**p value**	**p value**	**HR (95% CI)**	**p value**	**p value**	**HR (95% CI)**
sex	Male vs female	0.947	-		0.028	0.669	
age	≥66 vs <66	0.038	0.07		< 0.001	< 0.001	2.340 (1.556–3.497)
smoking history	ever vs never	0.934			0.019	0.068	
histologic subtype	PD vs. WD/MD	0.003	0.42		0.002	0.412	
pleural invasion	Present vs. absent	< 0.001			< 0.001	0.031	1.575 (1.042–2.379)
vascular invasion	Present vs. absent	< 0.001	0.026	1.444 (1.045–1.995)	< 0.001	0.693	
lymphatic invasion	Present vs. absent	< 0.001	0.003	1.667 (1.214–2.372)	< 0.001	0.006	1.885 (1.197–2.967)
Perineural invasion	Present vs. absent	0.04	0.665		0.009	0.301	
pTNM stage	IIB, III and IV vs I, IIA	<0.001	<0.001	2.129 (1.564–2.899)	< 0.001	< 0.001	2.684 (1.788–4.029)
PD-L1 protein expression	>1% vs <1%	0.196			0.054		
	>50% vs <50%	0.382			0.381		
PDL1 mRNA expression	positive vs negative	0.488			0.127		
**B (n = 340)**							
		**Disease-free survival**			**Overall survival**		
		**Univariate**	**Multivariate**		**Univariate**	**Multivariate**	
**Clinicopathologic variables**	**Category**	**p value**	**p value**	**HR (95% CI)**	**p value**	**p value**	**HR (95% CI)**
sex	Male vs female	0.475			0.13		
age	≥66 vs <66	0.749			0.021	0.043	1.830(1.020–3.284)
smoking history	ever vs never	0.501			0.01	0.017	2.026(1.133–3.624)
histologic subtype	PD vs. WD/MD	0.015	0.255		0.086		
pleural invasion	Present vs. absent	0.001	0.25		0.006	0.032	1.908(1.057–3.443)
vascular invasion	Present vs. absent	0.006	0.352		0.204		
lymphatic invasion	Present vs. absent	<0.001	0.069		0.001	0.02	2.038(1.120–3.709)
Perineural invasion	Present vs. absent	0.11			0.037	0.074	
pTNM stage	IIA vs. I	<0.001	0.008	2.323(1.361–3.966)	0.001	0.164	2.194(1.140–4.001)
PD-L1 protein expression	>1% vs <1%	0.129			0.02	0.05	1.947(1.000–3.791)
	>50% vs <50%	0.416			0.064		
PD-L1 mRNA expression	positive vs negative	0.887			0.909		

PD-L1, Programmed cell death ligand-1; mRNA, messenger RNA; HR, hazard ratio; CI, confidence interval; WD, well differentiated; MD, moderately differentiated; PD, poorly differentiated

## Discussion

In this study, we demonstrated that PD-L1 expression was significantly associated with histologic grade and genetic alteration status in lung ADC. We also found that PD-L1 protein expression was an adverse prognostic marker for OS in patients with early stage lung ADC. We also assessed *PD-L1* mRNA expression and compared PD-L1 protein and mRNA expression; the results showed that *PD-L1* mRNA was a potential surrogate marker, with a positive correlation with protein expression.

Our study showed that PD-L1 protein and mRNA expression levels were significantly associated with high histologic grade and solid subtype of ADC. Our results are in line with the results of several studies which reported the correlation between PD-L1 expression in tumor cells and poor differentiation and solid histology [9–12]. This finding may be clinically useful when small biopsies from patients show negative PD-L1 expression, but only the lepidic component was biopsied. Because small biopsies may miss the region of the tumor with high PD-L1 expression due to the heterogeneity issue [21], re-biopsy could be considered in solid tumor areas to ensure that the patient is a candidate. From our experience, PD-L1 was found to be strongly expressed in poorly differentiated cells but negative in papillary and lepidic components in a small biopsy specimen (not published data). In particular, in the case of pembrolizumab, which has received FDA approval as first line therapy for metastatic NSCLC, accurate evaluation of PD-L1 expression in advanced stage patients, which can only be performed with biopsy, is crucial for identifying patients to be a candidate to anti-PD-1 therapy [5, 22].

From biological point of view, elevated expression of PD-L1 poorly differentiated lung ADCs compared with well-differentiated ADCs might account the inactivation of effector-immune cells through PD-1 receptor signaling which could ultimately enhance tumor progression. This relationship supports the results of our study that ADCs with high expression of PD-L1 are associated with poorly differentiated histology and poor prognosis.

The relationship between *EFGR* mutation status and PD-L1 expression in NSCLC is still unclear. Although preclinical studies have suggested that EGFR-driven NSCLC inhibits antitumor immunity through activation of the PD-1/PD-L1 pathway in an intrinsic manner, epidemiological studies have suggested that *EGFR*-mutant NSCLC is more likely to exhibit decreased PD-L1 expression. Two recent pooled analyses have provided further support for this inverse relationship. In one study, patients harboring *EGFR* mutations were more likely to have decreased PD-L1 expression (odds ratio: 1.79, 95% confidence interval: 1.10–2.93) [23], and in another study, PD-L1 expression was associated with *EGFR* wild-type status (odds ratio: 0.61, 95% confidence interval: 0.42–0.90, *P* = 0.01) [24]. One reason for these conflicting results between *EGFR* mutations and PD-L1 expression could be the variability, including the assessment of biomarkers from a single lesion site at a single time point, which often provides poor insights into spatiotemporal dynamics. For example, PD-L1 expression has been shown to fluctuate during *EGFR* tyrosine kinase inhibitor (TKI) and post-progression [25]. In our study, none of the patients received *EGFR* TKI treatment, which could exclude the temporal heterogeneity of PD-L1 status. PD-L1 protein and mRNA expression were lower in the *EGFR*-mutated group than in the *EGFR*-negative group. Low PD-L1 expression in *EGFR*-mutated ADC may be related to the lower prevalence of PD-L1 in the Asian population than in Western populations. In our cohort, PD-L1 expression was observed in 16.2% of patients with a 1% cut-off and in only 6.1% of patients with a 50% cut-off. Several reports have shown low PD-L1 prevalence in lung ADC of Asian patients [26]. Thus, the difference in *EGFR* mutation prevalence may be one of the causes of ethnic differences in PD-L1 expression.

The prognostic impact on PD-L1 expression in NSCLC is still controversial [2,27], but our results along with several reports addressed the association of PD-L1 expression and poor clinical outcomes[11,28]. Koh et al. demonstrated that PD-L1 expression is a poor prognostic factor of DFS in patients with pulmonary ADC [11]. They suggested that PD-L1 expression in tumor cells and infiltration of PD-1+/CD8+ tumor infiltrating lymphocytes may not only induce T-cell exhaustion but also inhibit tumor cell death. A meta-analysis with 1,550 patients with NSCLC from nine studies has also demonstrated that PD-L1 protein expression in NSCLC is associated with poor prognosis [28]. Another meta-analysis reported that PD-L1 expression was associated with poor patient outcome in only Asian NSCLC subgroup, suggesting that ethnic difference might be associated with the prognostic implication of PD-L1 [29]. These discrepancies may be due to differences in the PD-L1 assay method, heterogeneity according to the NSCLC subtype, and various stages and treatment modalities [29]. To minimize these issues, we investigated the associations between PD-L1 expression and survival in a large cohort of patients with NSCLC, including many early stage tumors that had been resected with curative intention. Our study demonstrated that PD-L1 protein expression was a poor prognostic factor affecting overall survival in patients with early stage lung ADC, but had no prognostic value in patients with SqCC histology.

In this study, PD-L1 expression was found as frequently in stages I and II (24.8% and 27.8%, respectively) as in stages III and IV (23.4% and 24.0%, respectively), indicating that aberrant expression of this ligand may be an early event. Patients with early stage NSCLC may have a more intact immune system and the potential for long-lasting immune priming against micrometastases [30]. Therefore, immunotherapy for early stage cancer could increase the cure rate, reduce tumor burden, and enable local approaches (such as surgery) in additional patients, taking advantage of minimal residual disease. Ongoing clinical trials have been investigating the effects of neoadjuvant or adjuvant immunotherapy for resectable early stage lung cancer, and several studies have shown promising results. PD-L1 expression may be important not only as a prognostic factor but also as a predictive biomarker for early stage lung cancer immunotherapy. Thus, further studies are needed to determine whether PD-L1 may be related to drug response in early stage disease.

Finally, we assessed PD-L1 expression using IHC and RISH. Many studies have attempted to assess PD-L1 expression using different techniques, including RISH [15,16]. The OPA of RISH was over 80% compared with the FDA-approved IHC method, and our study illustrated the possible application of RISH as a complementary diagnostic test, providing accurate detection of PD-L1 in NSCLC. However, there was discrepancy in the expression of PD-L1 protein and mRNA. Among 68 cases with PD-L1 protein expression over 50% of tumor cells, 20 cases (29.4%) were confirmed no PD-L1 mRNA expression on RISH. There were several consideration of the discrepancy. First of all, RNA is a weaker molecule compared with DNA or protein, so RNA is more sensitive to procedures of fixation and deproteinization. Secondly, RISH is a semi-automatic procedure including the incubation step with the probe as a manual procedure, that could also affect the results. Although the novel RISH assay used in our study provide a higher level of target sensitivity and specificity when compared with many IHC protocols achieved using by Z-pairs oligonucleotides [19], there might be analytic variables, especially during manual procedures. Lastly, PD-L1 protein expression may be affect by post-transcriptional modification. Emerging evidence supports that PD-L1 expression is regulated on a post-transcriptional and translational level by various intracellular pathway [31,32]. These molecules can induce or suppress PD-L1 protein expression via PI3K/Akt signaling pathway or IFNγ pathway without *PDL1* mRNA expression. Further investigation of the mechanism of PD-L1 protein expression bypassing mRNA expression, and minimizing the analytic variables of RISH assay is necessary to reduce discrepancy between the two assay.

The lack of response data to anti-PD-1/PD-L1 drugs is the major limitation of our study. The issue on utilizing PD-L1 mRNA assay should be made based on data from clinical trials of the drug under consideration and further studies are needed with the therapeutic responses.

In conclusion, elevated PD-L1 expression was associated with poorly differentiated histology and *EGFR*-naïve status in lung ADC. In addition, PD-L1 expression may be a prognostic marker in patients with early stage lung cancer.

## Supporting information

S1 Table Supplementary table. (XLSX)
